# Assessment of HFE mutations in patients with iron overload

**DOI:** 10.1590/S1516-31802007000100014

**Published:** 2007-01-04

**Authors:** Paulo Lisboa Bittencourt

I read with interest the recently published manuscript from Cançado et al.^1^ concerning HFE genotyping of 35 Brazilian patients with iron overload. The authors had found five subjects homozygous for the C282Y mutation, four that had compound heterozygosity for C282Y/H63D and 17 subjects with at least one of the aforementioned HFE gene mutations. Sixteen of the patients had secondary causes of hemochromatosis^2,3^ due to hemolytic anemia, hepatitis C and alcoholic liver disease.

Based on these results, the authors described the presence of hereditary hemochromatosis in 10 patients (28%). They also reported a high frequency of C282Y/H63D heterozygotes (44%) in subjects with secondary iron overload, thus suggesting a role for HFE mutations in the development of iron overload in subjects with secondary hemochromatosis.

These findings should be interpreted with caution and some questions have to be raised in order to understand the role of HFE genotyping in patients with iron overload.

First, it is important to highlight that the authors have not defined any criteria for iron overload. Some of their patients had normal transferrin saturation and all had increased ferritin concentration. In addition, no information regarding liver biopsy was provided and it remains unknown whether those patients with hepatitis C or alcoholic liver disease would have been found to have siderosis on liver biopsy. It also has to be acknowledged that, to date, most patients with nonalcoholic steatohepatitis, hepatitis C and alcoholic liver disease who presented elevated transferrin saturation and ferritin levels have not had siderosis on liver biopsy.^2-4^

It was further reported that 10 (28%) of the patients in the study had hereditary hemochromatosis, on the basis of HFE typing. This assumption may be misleading, since the absence of HFE mutations does not rule out hereditary hemochromatosis.^2,3^ Indeed, approximately 40% of Brazilian patients with a well-defined phenotype of hereditary hemochromatosis lack classical HFE mutations, thus suggesting the occurrence of other HFE and non-HFE mutations in Brazilian subjects with hereditary hemochromatosis.^5^

Conversely, it was assumed that 10 patients in the study had hereditary hemochromatosis, on the basis of the presence of several different HFE genotypes. This could also be mislea ding, since liver biopsy is still necessary for diagnosing hereditary hemochromatosis in subjects who are not homozygous for the C282Y mutation,^3^ because of the frequency of these genotypes in the general population.^2,3^

Therefore, it has to be stressed that clear-cut criteria must be used to describe the phenotypes of hereditary hemochromatosis and secondary hemochromatosis. Patients with secondary hemochromatosis do not need to be HFE typed. On the other hand, HFE typing should be performed on subjects with primary iron overload characterized by elevated transferrin saturation and ferritin levels. In those iron-loaded subjects, homozygosity for the C282Y mutation leads to a diagnosis of hereditary hemochromatosis. Any other HFE genotype would require liver biopsy to assess the presence of siderosis. In geographical areas or in families where roles for other iron-loading genes have been revealed, mutations in other HFE and non-HFE genes could be contributory factors for a diagnosis of hereditary hemochromatosis.^3^

## References

[1] Cançado RD, Guglielmi AC, Vergueiro CS, Rolim EG, Figueiredo MS, Chiattone CS (2006). Analysis of HFE gene mutations and HLA-A alleles in Brazilian patients with iron overload. Sao Paulo Med J.

[2] Siah CW, Ombiga J, Adams LA, Trinder D, Olynyk JK (2006). Normal iron metabolism and the pathophysiology of iron overload disorders. Clin Biochem Rev.

[3] Tavill AS, American Association for the Study of Liver Diseases, American College of Gastroenterology, American Gastroenterological Association (2001). Diagnosis and management of hemochromatosis. Hepatology.

[4] Deguti MM, Sipahi AM, Gayotto LC (2003). Lack of evidence for the pathogenic role of iron and HFE gene mutations in Brazilian patients with nonalcoholic steatohepatitis. Braz J Med Biol Res.

[5] Bittencourt PL, Palacios SA, Couto CA (2002). Analysis of HLA-A antigens and C282Y and H63D mutations of the HFE gene in Brazilian patients with hemochromatosis. Braz J Med Biol Res.

