# Supplementary material for: Assessment of HFE mutations in patients with iron overload
Source: Sao Paulo Med J. 2007 Jan 4;125(1):65–7. doi: 10.1590/S1516-31802007000100014 (PMC11014702; doi:10.1590/S1516-31802007000100014)
Supplement: Related texts

# Reply: RESPONSE

**Authors:** Rodolfo Delfini Cançado

In 2002, an expert panel gathered to consider a new definition for hereditary hemochromatosis and at that time it was emphasized that “the disease of hereditary hemochromatosis is defined in terms of phenotype not genotype. Thus, the identification of any of the HFE mutations is, by itself, insufficient for the diagnosis of this disease. Instead, the identification of the genetic abnormality provides evidence of susceptibility to developing the phenotype”.^1^ In this regard Beutler wrote: “the HFE Cys282Tyr mutation is a necessary but not sufficient cause of clinical hereditary hemochromatosis”.^2^

Most cases (averaging 85%-90%) of hereditary hemochromatosis are caused by homozygosity for the C282Y mutation in the HFE gene. Another 3%-5% of patients with this disease are compound heterozygotes (C282Y/H63D).^3^

Patients with iron overload, particularly if they have environmental or other causes of iron accumulation and tissue injury (such as iron-loading anemia, chronic hemolysis, blood transfusions, excess dietary intake, hepatitis C, alcoholism, or steatohepatitis), should be considered to have acquired iron overload even if they are heterozygous for C282Y or H63D (found in 10%-20% and 15%-30% of persons of Northern European extraction, respectively).^3^

In our study, the criteria for diagnosing iron overload included two fasting measurements of the transferrin saturation index (TS), with results ≥ 60% in males and ≥ 50% in females, and/or serum ferritin con centrations exceeding 300 ng/ml in males or 200 ng/ml in females.

According to Powell (2002), the criteria for diagnosing established hereditary hemochromatosis include at least one of the following in the absence of other known causes of iron overload: 1) Stainable hepatic iron grade 3 or 4; 2) Hepatic iron concentration > 80 mM/g (dry weight); 3) Hepatic iron index (hepatic iron concentration/age) > 1.9; 4) Removal of 5 g of iron or more by therapeutic phlebotomy without inducing iron deficiency; 5) Diagnosis of HFE-associated hemochromatosis, confirmed by demonstration of homozygosity for the C2828Y mutation or compound heterozygo sity for C282Y and H63D.^4^

In our study, 12 out of 35 iron overload patients underwent liver biopsy. All of them presented hepatic siderosis (grade 3 or 4 stainable hepatic iron). Their main clinical and laboratory characteristics are shown in Table 1.

**Table 1.** Main clinical and laboratory characteristics of 12 Brazilian patients with grade 3 or 4 hepatic siderosis from liver biopsy

Age (years)	Gender	Genotype	Transferrin saturation index (%)	Serum ferritin ng/ml	Hepatitis C	Hepatic cirrhosis	Excessive alcohol consumption	Chronic hemolytic anemia
57	M	C282Y/C282Y	66.0	4218.0	No*	No	No	No
55	M	C282Y/C282Y	77.0	2200.0	No*	No	No	No
59	F	C282Y/C282Y	83.0	1265.0	No†	Yes	No	No
54	F	C282Y/C282Y	75.0	1883.0	No*	No	No	Yes*
67	F	C282Y/C282Y	85.0	5265.0	No†	Yes	No	No
54	F	C282Y/H63D	66.0	2324.0	No†	Yes	No	No
67	M	C282Y/H63D	57.0	1359.0	No*	No	No	Yes*
47	F	C282Y/WT	67.0	324.0	Yes*	No	No	No
54	M	C282Y/WT	68.0	1200.0	No*	No	No	No
42	F	H63D/WT	57.0	2500.0	No*	No	No	Yesj
44	M	H63D/WT	55.0	1573.0	Yes*	No	No	No
51	M	WTWT	65.0	1180.0	Yes*	No	Yes	No

M = male; F = female; WT (wild type) = Normal;

* = hereditary spherocytosis;

† = alpha thalassemia.

In some patients with secondary hemochromatosis, it is important to study the HFE mutations, especially in those who present iron overload much greater than expected.

Heterozygosity for C282Y or H63D may contribute towards iron overload because of another condition, but should not be considered to be the only cause of iron overload or as diagnostic for hereditary hemochromatosis.

Bacon et al. observed that in many chronic liver diseases, heterozygosity of mutations in HFE may contribute to iron overload.^3^ We observed that the beta-thalassemia trait might increase the severity of hemochromatosis in subjects with the C282Y mutation in the HFE gene.^5^ Piperno et al. demonstrated that the beta-thalassemia trait aggravated the clinical condition of C282Y homozygotes, thus favoring higher rates of iron accumulation and the development of severe iron-related complications. They suggested that the coexistence of the beta-thalassemia trait might also increase the risk of iron overload in patients with HFE genotypes who were at mild risk of hemochromatosis.^6^

We agree with Bittencourt PL that the absence of HFE mutations does not rule out hereditary hemochromatosis. We know that there are other hereditary forms of this disease that may be secondary to other mutations in the HFE gene or other genes involved in iron metabolism. We are now studying a mutation in the transferrin receptor-2 gene and in the DMT-1 gene, among patients with iron overload and normal HFE genotype.

## References

[1] McCullen MA, Crawford DH, Hickman PE (2002). Screening for hemochromatosis. Clin Chim Acta.

[2] Beutler E (2003). The HFE Cys282Tyr mutation as a necessary but not sufficient cause of clinical hereditary hemochromatosis. Blood.

[3] Bacon BR, Powell LW, Adams PC, Kresina TF, Hoofnagle JH (1999). Molecular medicine and hemochromatosis: at the crossroads. Gastroenterology.

[4] Powell LW (2002). Hereditary hemochromatosis and iron overload diseases. J Gastroenterol Hepatol.

[5] Arruda VR, Agostinho MF, Cançado R, Costa FF, Saad ST (2000). Beta-thalassemia trait might increase the severity of hemochromatosis in subjects with the C282Y mutation in the HFE gene. Am J Hematol.

[6] Piperno A, Mariani R, Arosio C (2000). Haemochromatosis in patients with beta-thalassaemia trait. Br J Haematol.
